# Cancer microcell initiation and determination

**DOI:** 10.1186/s12885-021-08813-5

**Published:** 2021-10-08

**Authors:** Zane Simsone, Tālivaldis Freivalds, Dina Bēma, Indra Miķelsone, Liene Patetko, Juris Bērziņš, Līga Harju, Indulis Buiķis

**Affiliations:** 1 Institute of Cardiology and Regenerative Medicine, University of Latvia, Jelgavas Street 3, Riga, LV-1004 Latvia; 2 Institute of Clinical and Preventive Medicine, University of Latvia, Gailezera Street 1, Riga, LV 1079 Latvia; 3 Department of Human Physiology and Biochemistry, Rīga Stradiņš University, Dzirciema Street 16, Riga, LV-1007 Latvia; 4 Laboratory of Bioanalytical and Biodosimetry Methods, Faculty of Biology, University of Latvia, Jelgavas Street 3, Riga, LV-1004 Latvia

**Keywords:** Microcell, cancer, cancer resistance, Paclitaxel, Doxorubicin, Cell viability, NADPH, SK-MEL-28

## Abstract

**Background:**

Cancer remains one of the leading causes of death worldwide, despite the possibilities to detect early onset of the most common cancer types. The search for the optimal therapy is complicated by the cancer diversity within tumors and the unsynchronized development of cancerous cells. Therefore, it is necessary to characterize cancer cell populations after treatment has been applied, because cancer recurrence is not rare. In our research, we concentrated on small cancer cell subpopulation (microcells) that has a potential to be cancer resistance source. Previously made experiments has shown that these cells in small numbers form in specific circumstances after anticancer treatment.

**Methods:**

In experiments described in this research, the anticancer agents’ paclitaxel and doxorubicin were used to stimulate the induction of microcells in fibroblast, cervix adenocarcinoma, and melanoma cell lines. Mainly for the formation of microcells in melanoma cells. The drug-stimulated cells were then characterized in terms of their formation efficiency, morphology, and metabolic activity.

**Results:**

We observed the development of cancer microcells and green fluorescent protein (GFP) transfection efficiency after stress. In the time-lapse experiment, we observed microcell formation through a renewal process and GFP expression in the microcells. Additionally, the microcells were viable after anticancer treatment, as indicated by the nicotinamide adenine dinucleotide hydrogen phosphate (NADPH) enzyme activity assay results. Taken together, these findings indicate that cancer microcells are viable and capable of resisting the stress induced by anticancer drugs, and these cells are prone to chemical substance uptake from the environment.

**Conclusion:**

Microcells are not only common to a specific cancer type, but can be found in any tumor type. This study could help to understand cancer emergence and recurrence. The appearance of microcells in the studied cancer cell population could be an indicator of the individual anticancer therapy effectiveness and patient survival.

**Supplementary Information:**

The online version contains supplementary material available at 10.1186/s12885-021-08813-5.

## Background

Cancer is a major human health problem and remains the second leading cause of death worldwide, after cardiovascular disease [1]. In the year 2018 alone, there were 18.1 million new cancer incidences worldwide, with more than half of all cases being lethal [2]. Tumor heterogeneity and cancer cell polymorphism and differentiation in various developmental stages in the same tumor make it very difficult to choose the most efficient and suitable therapy. While significant efforts have already been made to characterize cancer cell population within untreated tumors, cancerous cell population after applied chemotherapy has not been understood. Buiķis and colleagues have previously shown that human sarcoma cell cultures treated with thiophosphamide (Thio-TEPA), a chemotherapeutic agent used to treat breast, ovarian and bladder cancers [3, 4] exhibit the development of unusual small-sized cells, called microcells. These cells are round or oval, with a small amount of cytoplasm, and are intensively stained by bromo-2-deoxyuridine (BrdU) that is thymidine analog and it incorporates into DNA and shows cells in S-phase and methyl green-pyronine that stains nucleic acids both in DNA and RNA [4–6]. A population of microcells has also been observed in triple-negative breast cancer histological samples obtained from patients [7]. Microcells from patient histological samples stained with Feulgen assay are characterized by a small nucleus and a high average optical density, which is proportional to the amount of DNA in a cell [7]. Microcells have been described as a natural tumor component, but interestingly their relative numbers increase after cancer therapy has been initiated. Buiķis et al. [8] have proposed a hypothesis explaining the process of microcell development by sporosis, which is a process of microcell formation from a damaged tumor macrocell [8]. Indeed, it has been shown that the number of viable microcells rapidly increases in tumor tissues after anticancer therapy has been initiated. One defective macrocell may produce one or several microcells [4, 8].

Microcells are viable and appear to resist cancer treatment. Thus, it is of a great importance to characterize this cell subpopulation, in order to develop new treatment strategies and improve therapy efficiency. In this work, we have identified and morphologically characterized microcells and their formation after initiation of anti-cancer treatment with doxorubicin and paclitaxel. Doxorubicin (DOX) and paclitaxel (PTX) are the most effective drugs currently used in anticancer therapy of various types of cancers, for example breast, ovarian, lung cancers and melanoma [9–11]. DOX is a widely used anticancer drug that intercalates within DNA base pairs, causing damage to DNA strands, inhibiting both DNA and RNA synthesis and thereby arresting cell proliferation [11, 12]. PTX, on the other hand, is an antimicrotubular agent. PTX causes abnormal stabilization of dynamic microtubule polymerization, leading to the failure of mitosis, proliferation, invasion, and cancer cell colony formation [10, 13, 14]. In this study we have identified microcell formation in three different cell lines: human melanoma (SK-Mel-28), human skin fibroblast (HS-68) and cervical carcinoma (HeLa) cells. Furthermore, using 8-anilinonaphthalene-1-sulfonic acid (ANS)- Ethidium bromide (EtBr), nicotinamide adenine dinucleotide hydrogen phosphate NADPH assay, and time-lapse experiments with green fluorescent protein (GFP) transfection, we have shown that the microcells are viable and metabolically active. Several studies report observation of cells which from after chemotherapy; these cells are metabolically active cells, with high proliferation activity and increasing NADPH, which improves cell viability and aging escape [15–17]. Various studies focus on drug-resistant cell populations in aggressive types of cancer [18–20], but without morphological characterization of these cells. Melanoma is the most aggressive skin cancer, with poor response to treatment, with a melanoma cell line study showing DOX chemoresistance ability [21]. SK-MEL-28 is a DOX- and PTX-resistant human melanoma cell line [6, 22, 23].

## Methods

### Cell lines

Human cervical carcinoma (HeLa), SK-MEL-28 melanoma, and HS-68 (human skin fibroblast) cell lines were acquired from the American Type Culture Collection (ATCC) and maintained in a culture medium consisting of Dulbecco’s modified Eagle’s medium (DMEM; Thermo Scientific, IL, USA) supplemented with 10% fetal bovine serum (FBS). All cell lines were seeded as monolayer on cover glass into 24-well plates with an initial density of ~ 1 × 10^5^ cells per well and grown in a humidified atmosphere containing 5% CO_2_ at 37 °C. DMEM supplemented with 10% FBS and 10 mg/mL of a penicillin/streptomycin solution (all from Sigma-Aldrich, MO, USA).

To our knowledge, the microcells were so far only observed in human sarcoma (HT-1080) and human cervical carcinoma (HeLa) cell lines and Djungarian hamster fibroblastomas cell line 4/21 [4, 8, 24]. Therefore, we used three cell lines to determine if microcells could be forming in other cancer (HeLa, SK-MEL-28) and non-cancer (HS-68) cell lines. In this study we used the HeLa cell line, to which is known that microcells formation is initiated by applied stress factors. The second used cell line is a human fibroblast cell line (HS-68) described as normal cells, and the third cell line is melanoma cells which are quite aggressive skin cancer.

### Stress factors

Stress factors were applied to cancer microcell formation. Cell lines HeLa, HS-68, and SK-MEL-28 were treated for 24 h with doxorubicin (DOX; 50 mg; TEVA) at a final concentration of 2.5 μM or with paclitaxel (PTX; 6 mg/mL; TEVA) at a final concentration of 0.7 μM at 37 °C in a 5% CO_2_ atmosphere. The DOX and PTX were not used together. After treatment, the DMEM was replaced with fresh medium, and the cells were cultivated for another 24 and 48 h at 37 °C in a 5% CO_2_ atmosphere.

The HeLa cell line was treated with methanol (Sigma Aldrich, USA) at final concentration 7.5% for 1 h at 37 °C in 5% CO_2_ atmosphere. After the treatment, cells were cultivated in fresh DMEM medium for 6 h. The cultivated cells were fixed with 4% formaldehyde solution for 10 min at room temperature.

### NADPH test for cell metabolic activity

Nicotinamide adenine dinucleotide hydrogen phosphate-diaphorase (NADPH-d) is an enzyme that can reduce nitro blue tetrazolium (NBT) dye to the visible reaction product formazan [25]; the reaction involves hydrogen transfer from the substrate at presence of NADPH, to a hydrogen acceptor. Initially, SK-MEL-28 cells were cultivated for 24 h with 0.7 μM of PTX, then the medium was changed and the cells were cultivated for another 24 h. The NADPH test started with pre-fixation for 1 min with a 4% formaldehyde solution (Sigma-Aldrich, MO, USA), then cells were carefully washed two times with 1.5 M TRIS buffer saline and incubated for 30 min in the incubation medium (2.5 mL of 1.5 M TRIS buffer saline, 1.5 g of sucrose, 0.5 mL of nitro blue tetrazolium (Sigma-Aldrich, MO, USA), and 0.9% NaCl to 10 mL). NADPH substrate (β-Nicotinamide adenine dinucleotide phosphate reduced sodium salt hydrate; SERVA, Germany) was administered to the solution shortly before incubation. The reaction was stopped with a 4% formaldehyde solution fixation for 10 min at room temperature, washed with distilled H_2_O and covered with coverslips using CV ultra-mounting medium (Leica Biosystems, Nussloch, Germany). NADPH diaphorase activity was seen as dark blue or black spots in the cell cytoplasm. The NADPH activity was scored semi-quantitatively by two independent observers using ZEISS microscopy Camera Axiocam 202 mono, where 0 points- activity was not detected; 1 point- low activity; 2 points- activity was detected in average level; 3 points high activity, but not in a whole cell or sample; 4 points strong activity in the entire cell or sample.

### ANS-ethidium bromide staining

The 8-anilinonaphthalene-1-sulfonic acid (ANS) – ethidium bromide (EtBr) staining technique was used to detect presence of proteins and nucleic acids in cells. ANS is a staining protein at hydrophobic site of a protein, it fluoresces in a blue light [26, 27], while EtBr fluoresces in a red light, thereby revealing DNA and RNA [28].

HeLa cells were seeded in monolayers on a coverslip at a density of ~ 1 × 10^5^ cells per well and grown in a humidified atmosphere 1containing 5% CO_2_ at 37 °C. DMEM growth medium was supplemented with 10% fetal bovine serum and 10 mg/mL of a penicillin/streptomycin solution (all from Sigma Aldrich, USA). When cell density was 80–100% on the monolayer, a final concentration of 7.5% methanol (Sigma-Aldrich, MO, USA) was added. Cells were incubated for 1 h at 37 °C in a 5% CO_2_ atmosphere. After treatment, the cells were cultivated in fresh DMEM for 6 h. The cultivated cells were fixed with a 4% formaldehyde solution for 10 min at room temperature, then stained with 5 μg/mL of ANS (Sigma-Aldrich, MO, USA). The dye solution was kept in the dark, poured the cover slide with cells, incubated for 10 min at room temperature, before being drained without washing. EtBr (3 μg/mL; Sigma-Aldrich, MO, USA) was poured on the cover slide with the cells, incubated for 10 min at room temperature, kept in the dark, and then drained without washing. The cells were dried in the air, and covered with coverslips using CV ultra-mounting medium (Leica Biosystems, Nussloch, Germany). ANS–EtBr fluorescence was visualized using a three-band blue, red, and green (Leica BRG) optical filter and a Leica DM1000B microscope qualitatively scored blinded by two independent observers as negative or positive staining.

### The neutral red uptake assay (NRU)

Neutral red uptake assay is used for cytotoxicity detection. The principle of this assay is based on the detection of viable cells via the uptake of the dye neutral red [29]. SK-MEL-28 cells were seeded into the 24-well plate on the coverslips at the concentration of ~ 1 × 10^5^ cells per well and grown in a humidified atmosphere containing 5% CO_2_ at 37 °C. Reaching 80% of confluency cells were treated with paclitaxel (PTX; 6 mg/mL; TEVA) at a final concentration of 0.7 μM at 37 °C in a 5% CO_2_ atmosphere for 24 h. After the treatment, the DMEM was replaced with fresh medium, and the cells were cultivated for another 24 at 37 °C in a 5% CO_2_ atmosphere. Sequentially neutral red (NR, Sigma-Aldrich, Taufkirchen, Germany) was added to control and treated cells for 3 h incubation. After 3 h the staining solution was removed, cells were rinsed three times with PBS. Then cells were fixed with 4% formaldehyde solution for 10 min in room temperature and rinsed three times with PBS. Coverslips were removed from the 24-well plate coated with CV ultra-mounting medium and analyzed under a microscope.

### Cell transfection and GFP expression time lapse

Initially, SK-MEL-28 cells were seeded on glass-bottom 24-well plates (Cellvis, CA, USA) at a concentration of ~ 1 × 10^5^ cells per well and grown in a humidified atmosphere containing 5% CO_2_ at 37 °C. DMEM cultivation medium was supplemented with 10% fetal bovine serum and 10 mg/mL of a penicillin/streptomycin solution (all from Sigma-Aldrich, MO, USA). When the SK-MEL-28 -melanoma cells reached 70–80% confluence, the medium was replaced with fresh growth medium containing 10% FBS (Sigma-Aldrich, MO, USA).

A transfection mix for one sample was prepared using 1 μg of plasmid pcDI-EGFP (6837 bp) containing GFP gene diluted in 100 μL of serum-free DMEM with an additional 2 μL of TurboFect™ reagent (Thermo Scientific, IL, USA), according to the manufacturer’s protocol [30]. A plasmid pcDI-EGFP allows GFP expression in viable cells. The plasmid and TurboFect™ complex were incubated for 15 min at room temperature. Then, 100 μL of the TurboFect™/plasmid mixture drop was used to transfect each well of the plate. The cells were grown for 24 h in the presence of the plasmid; after the incubation period, the growth medium was replaced with DOX (50 mg; TEVA) at a final concentration of 2.5 μM into each well, and cultivated for 24 h at 37 °C in 5% CO2, normoxia, using a confocal laser microscope (Leica SP8 Confocal, Leica, Wetzlar, Germany) cell cultivation chamber. Then the medium was changed for a fresh medium without DOX.

The confocal laser microscope transmitted light and the fluorescent light detectors were equipped at the argon laser line of 488 nm, the time lapse function was used for time lapse imaging. Images were taken every 120 min after doxorubicin administration and examined for 72 h. GFP was excited by 488 nm laser line and the emission was detected at 510 nm.

### TSG101 antigen expression detection

SK-MEL-28 cells were cultivated on coverslips at the concentration of ~ 1 × 10^5^ cells per well and grown in a humidified atmosphere containing 5% CO_2_ at 37 °C. Reaching 80% of confluency cells were treated and microcell formation induced with PTX at a final concentration of 0.7 μM at 37 °C in 5% CO_2_ atmosphere for 24 h. After the treatment, the DMEM was replaced with fresh medium, and the cells were cultivated for another 24 at 37 °C in a 5% CO_2_ atmosphere. Then cells were fixed with 4% formaldehyde solution for 10 min in room temperature and rinsed with PBS two times for 5 min and antigen retrieval was done with 0.2% Triton X-100. After that samples were rinsed with PBS three times for 5 min and one time with PBS/Tween 20 for 5 min. Sequentially samples were blocked with 2% BSA/PBS for 1 h in room temperature. Blocking solution was decanted without washing and added TSG101 (GTX635396; GenTex, NordicBioSite, Finland) monoclonal rabbit anti-human primary antibody diluted 1:50 in 1% BSA/PBS, and incubated overnight at 4 °C in a humidified chamber. Samples were then washed trice with PBS for 5 min and once with PBS/Tween 20 for 5 min and covered with Alexa fluor 488 (ab150077, Abcam, CA, USA) goat anti-rabbit secondary antibody diluted 1:200 in 1% BSA/PBS, and incubated for 1 h in a humidified chamber in the dark. Samples were washed trice with PBS for 5 min and once with PBS/Tween 20 for 5 min. Cell nuclei were counterstained with 1 μg/mL DAPI for 1 min and finally coated with CV ultra-mounting medium and analyzed under a microscope.

### Determination of cells number

The cells count was determined using a Leica DM1000B microscope (Leica Microsystems) with a 40× objective (dry, plan apochromatic, with a numeric aperture of 0.85). The microcell count and the whole number of HeLa, HS-68, and SK-MEL-28 cells in the three experiments in at least ten fields of view were obtained. The total number of counted cells was 1060 cells at each time point: 24, 48, and 72 h after applied therapy.

### Microscopy and image analysis

A Leica DM1000B microscope (Leica Microsystems, Wetzlar, Germany) with 63× objective (oil, plan apochromatic, with a numeric aperture of 1.40) equipped with a Leica DFC400 (Leica Microsystems, Wetzlar, Germany) digital camera and ZEISS Axiolab 5 microscope with 63×/0.85 objective (oil, N-Achroplan, Ph3 M27; ZEISS, Jena, Germany) with ZEISS Microscopy Camera Axiocam 202 mono (ZEISS, Jena, Germany) were used for microscopy of the samples. For image analysis and processing, Image-Pro**®** plus the Proven Solution™ software, version 4.0, and LAS X lite (Leica Microsystems, Wetzlar, Germany) and ZEN 3.0 Blue lite (ZEISS, Jena, Germany) were used.

### Transmission Electron microscopy

Transmission electron microscopy was done in previous pilot experiments inducing microcells by stress factors. In our study we decided to use the archive materials of TEM. The cell line 4/21 was seeded in 24 cm2 Karell flasks with a density of ~ 3 × 10^5^ cells per well and grown in Eagle medium supplemented (Sigma-Aldrich, MO, USA) with 10% FBS and 1% penicillin/streptomycin (growth medium) in a humidified atmosphere containing 5% CO_2_ at 37 °C. Cells were treated with Thio-TEPA at a final concentration 20 μg/mL for the 24 h. After treatment, the medium was removed and washed with medium without serum, and fresh growth medium was added and incubated in for the 24 h. The incubation medium was removed and 4 °C 2.5% glutaraldehyde solution prepared in PBS at 4 °C was added for the cell sample preparation for electron microscopy. The cells were fixed for 15 min in the 2.5% glutaraldehyde solution. Sequentially the cell monolayer was detached gently using a cell scraper and collected in a test tube, and centrifugated for 10 min at 200 g. Then cells were washed twice with PBS at 4 °C and postfixed with 2% osmium acid solution prepared in PBS for 10 min. The pellet was dehydrated in 70% ethanol and embedded in Epon (Epoxy embedding medium; Sigma-Aldrich,MO, USA). Ultrathin sections were contrasted by uranylacetate and lead citrate, and examined using an electron microscope JEM-100B (TEM, Japan).

### Statistical analysis

The number of microcells in the control (untreated) and treated cells was compared using two-tailed Student’s t-test for unpaired samples was used. Statistical significance of difference of means were calculated where appropriate. The differences were considered significant at *p* ≤ 0.05. The percentages were counted, assuming a total cell count of 1060 being 100%.

## Results

### Initiation of microcell formation using paclitaxel and doxorubicin

The microcells were previously observed in human sarcoma (HT-1080) and human cervical carcinoma (HeLa) cell lines [4, 8]. The HeLa cell line was chosen as cell line to which is known that microcells formation is initiated by applied stress factors, such as chemotherapeutic drugs Thio-TEPA [4]. Therefore, to prove that microcells development is not specific to one cell line type, we used three different cell lines were used to determine if the microcells formed in other cancer and non-cancer cell lines.

In this study, we used the HeLa cell line, and microcells were initiated by applying DOX, PTX or methanol as stress factor. Additionally, the human fibroblast cell line HS-68, described as normal cells, and the melanoma cell line, which is quite an aggressive skin cancer, were used [23].

**Fig. 1:**
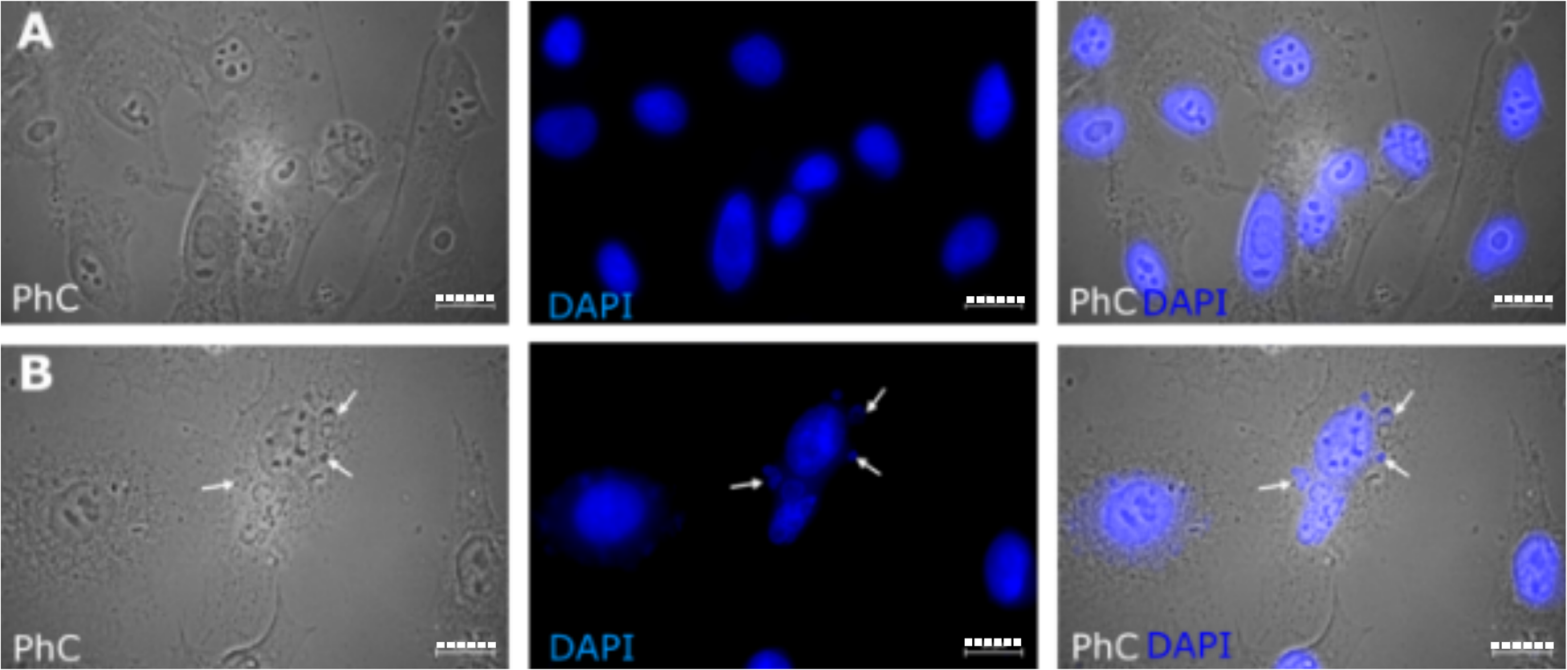
Microcell formation in SK-MEL-28 cells after paclitaxel (PTX) treatment for 48 h. (**A**) Untreated SK-MEL-28 cells (control sample) and phase contrasts (PhC); cell nuclei are stained with DAPI (blue) and are overlaid (PhC/DAPI). (**B**) SK-MEL-28 PTX-treated cells, PhC. The white arrows show the microcell formation. The cell nuclei are stained with DAPI (blue) and are overlaid (PhC/DAPI). The scale bar is 20 μm

After the SK-MEL-28 cell cultivation for 48 h following chemotherapy (in this case, treatment with PTX), the cell samples were examined (Fig. 1). The control sample (untreated) cells were spindle-shaped with a round and elongated nucleus (Fig. 1A). We observed small, round, and intensively stained cells, which formed after PTX therapy in the SK-MEL-28 cell line (Fig. 1B). In contrast, when therapy was not used, microcells were not seen at all (Fig. 1A). The increasing number of microcells shows the cell’s ability to survive the impact and effects of medication.

**Fig. 2:**
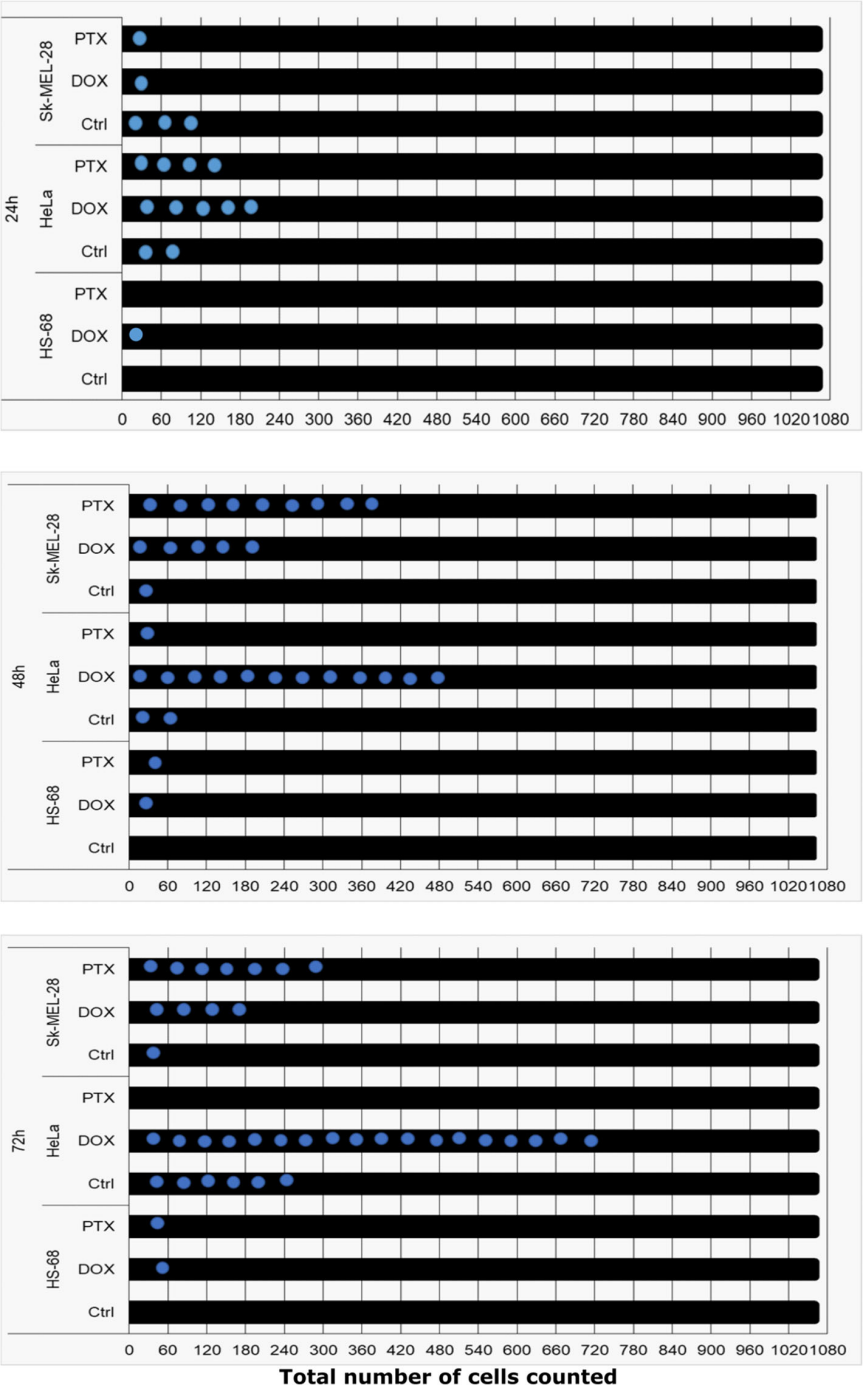
Increase in the number of microcells after applied therapy. Cells were counted after 24, 48, and 72 h following doxorubicin (DOX) and paclitaxel (PTX) treatment. The total number of counted cells was 1060 cells Microcells—blue dots representing each individual cell out of the 1060 counted cells. In HS-68, the fibroblasts cells, the increase in the number of microcells was small: One of the 1060 cells after treatment. Only in the cancer cell lines (HeLa and SK-MEL-28) were microcells evaluated in the control (Ctrl) untreated cells and forty-eight hours after applying PTX and DOX, there was a tendency for the microcells count increase

The 1060 cells were counted in this study in each of the used cell lines— HeLa, HS-68, and SK-MEL-28 cells. In the used HeLa and SK-MEL-28 cell lines under investigation, the microcells (shown as blue dots in Fig. 2) were mostly observed after anticancer treatment with PTX and DOX (72 h; Fig. 2). The microcells showed an increasing tendency in the cell population of the HeLa cell line. The highest number of microcells was observed 72 h after DOX treatment (18 microcells out of 1060 cells) in the HeLa cell line (Fig. 2): 1.57% from all counted cells, and the percentage increase was statistically significant (Table 1). Interestingly, the number of HeLa microcells after PTX treatment decreased, as compared to 48 h post-treatment. SK-MEL-28, the human skin melanoma cells, were treated with PTX. The largest number of microcells in SK-MEL-28 (nine microcells out of 1060 cells) was observed after 48 h after PTX treatment (Fig. 2), which is up to 1% (*p* < 0.016) of the total cell count (Table 1). In the fibroblast cell line (HS-68), no microcells were observed as expected. After applied treatment with DOX or PTX, the increase in the number of microcells in non-cancerous HS-68 cell line was small: One of the total counted cells. Results of this experiment indicate that microcells are natural components of a tumor, as these cells were detectable in untreated cancer cells and the number of microcells increase in both cervical carcinoma and melanoma cells, following DOX or PTX treatment. However, it appears that microcell formation in cancerous cell lines is stimulated by applied chemotherapy, as microcell count increases significantly after treatment with DOX or PTX, compared to non-cancerous HS-68 cell line.

**Table 1:** Microcells count 24, 48 and 72 h after DOX (Doxorubicin) and PTX (Paclitaxel) treatment in HS-68, HeLa and SK-MEL-28 cell lines

Cell Line	24 h					48 h					72 h				
	Ctrl (%)	DOX (%)	***p***-Value	PTX (%)	***p***-Value	Ctrl (%)	DOX (%)	***p***-Value	PTX (%)	***p***-Value	Ctrl (%)	DOX (%)	***p***-Value	PTX (%)	***p***-Value
HS-68	NA	0.09	0.324	NA	NA	NA	0.09	0.324	0.09	0.324	NA	0.09	0.324	0.09	0.324
HeLa	0.28	0.46	0.437	0.37	0.684	0.19	1.11	**0.013**	0.09	0.560	0.56	1.57	**0.047**	0.00	**0.023**
SK-MEL-28	0.28	0.19	0.700	0.09	0.302	0.09	0.65	**0.037**	0.83	**0.016**	0.09	0.37	0.157	0.56	0.151

Significant microcell count increase *p* values (≤ 0.05) are highlighted in bold. p values obtained by Student’s t test. *Ctrl* control, *NA* no microcells detected

### Proteins and nucleic acids in early formed microcells

**Fig. 3:**
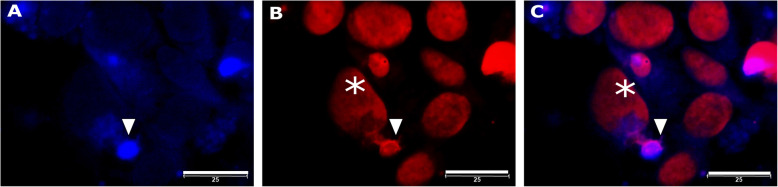
Microcell formation in the HeLa cell line after treatment with 7.5% methanol. (**A**) Microcell formation (blue protein body (ANS), white arrow); (**B**) an EtBr-stained microcell (white arrow) in contact with a macrocell (white star); (**C**) overlay (**A**, **B**). ANS–EtBr staining 6 h after treatment. The scale bar is 25 μm

ANS-EtBr staining method was used to identify proteins and nucleic acids in the microcell. ANS stains proteins – shown in blue and EtBr stains nucleic acids – stains red. Methanol stimulates apoptosis, and it was used to compare if the microcell formation observed as well as after chemotherapeutic drugs in HeLa cell line (known that microcells formatting). At first, microcells were observed after 6 h of exposure to 7.5% methanol (Fig. 3), which had a toxic effect on HeLa cell viability. ANS dye and ethidium bromide (EtBr) were used to identify presence of the proteins and nucleic acids in the microcells (Figs. 3). As shown in Fig. 3A, macrocells contain a smaller number of proteins compared to microcells. This indicates that the protein synthesis in macrocells is weak. When the microcells segregated from the macrocells, the microcells contained proteins (Fig. 3A, C, white arrow) and a small amount of nucleic acid (Fig. 3B, C). Although the microcells contained both proteins and nucleic acid, this is not yet indicated that the cells are viable. Therefore, it is important to research therapy surviving cells, determining the functional properties and markers characterizing microcells.

### Microcell formation and GFP expression after doxorubicin therapy: time lapse experiment

ANS-EtBr staining experiment has shown that the microcells contained both proteins and nucleic acids, however it was not clear if these cells are viable and can proliferate. Therefore, we set to determine if microcells are alive and able to express proteins. We have used GFP expression as a marker for cell viability. For following experiments, we used the SK-MEL-28 cell line to looking forward microcell formation in the one of the most aggressive skin cancers, melanoma.

**Fig. 4:**
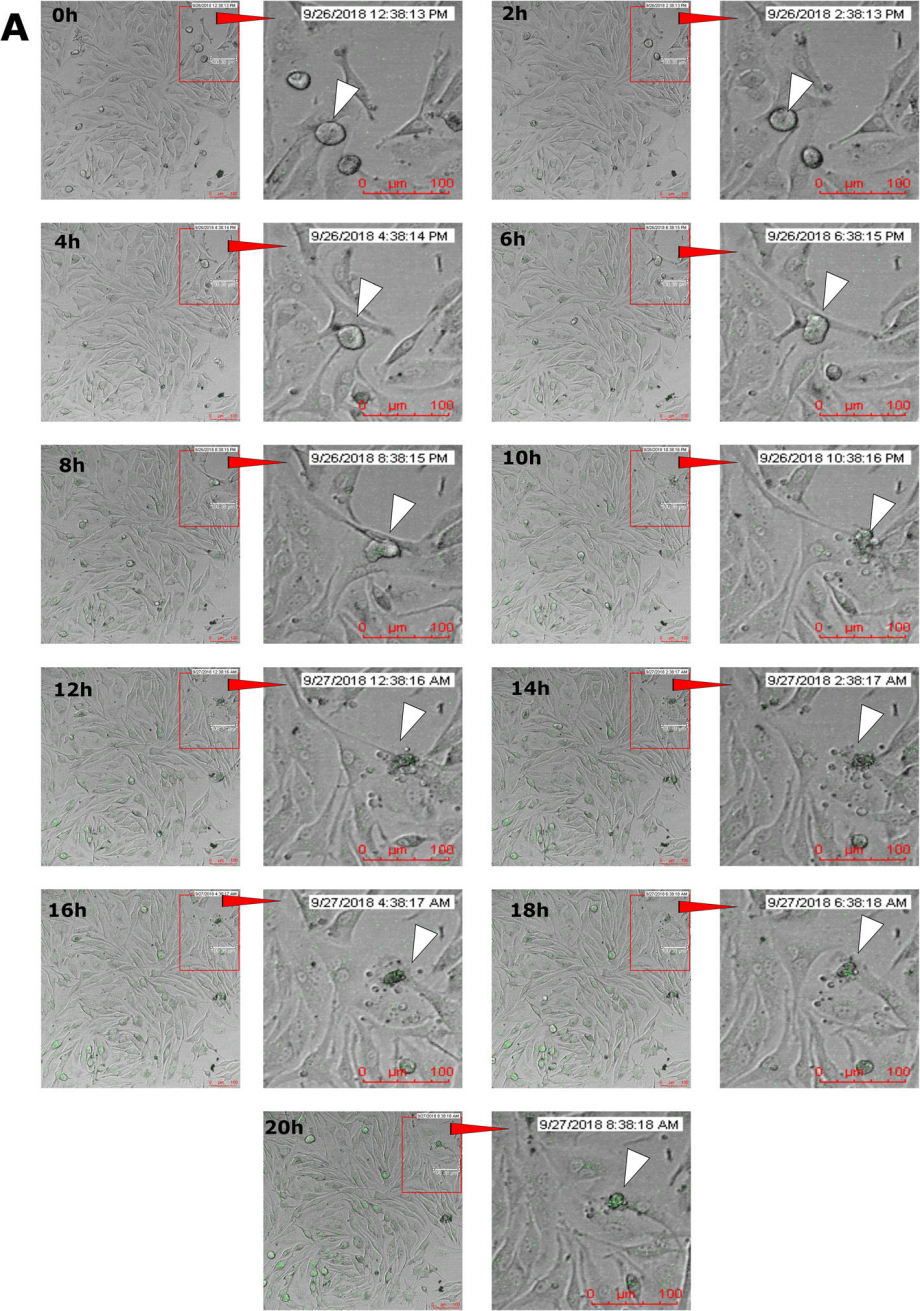
Microcell development and GFP expression after 24 h doxorubicin (DOX) treatment in SK-MEL-28 cells. The images were taken every 2 h; the red squares mark the region of interest (ROI). At 0, 2, 4, 6, 8, and 10 h, the cell was rounded, looking morphologically similar to the beginning of apoptosis (white arrow); at 12, 14, 16, and 18 h, the start of development of microcells (16–18 h, white arrow) and the expression of EGFP could be observed. At 20 h (white arrow), microcells were fully developed and expressed GFP. The scale bar is 100 μm


The 72 h time lapse experiment (see Materials and Methods for details) showed that 24 h after chemotherapy, the cancer cell morphologically behaved similar to apoptotic cells. Specifically, cell was rounded (Fig. 4), their cytoplasm produced blebs (10 h; Fig. 4), and the cell formed apoptotic-like bodies (12–14 h; Fig. 4). However, every 2 h in time lapse images following this, we observed that a microcell categorized as an apoptotic cell was capable of self-regeneration. This process appears to be visually similar to active endocytosis, when a cell is hosting particles from the extracellular space and regenerates (transforms) into a microcell. Herein, the microcells displayed the following morphological features: roundish and small-sized cells (approximately 3–5 μm) with endocytic ability and, finally, GFP expression (16–20 h; Fig. 4; see Supplementary file 1), indicating viability and activity. The results of this experiment showed that only a part of total cell population expresses GFP, however microcells are also capable of GFP expression, which indicates that these cells have retained the necessary machinery for protein expression and thus are viable and metabolically active.

**Additional file 1: Supplementary file 1:** GFP expression in the microcell after 48 h doxorubicin treated SK-MEL-28 cells. Leica SP 8 confocal microscope, objective 63x.

### Detection of the microcell metabolic activity using NADPH test

**Fig. 5:**
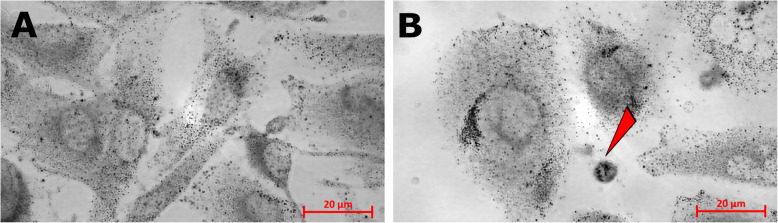
Microcell metabolic activity determined by a NADPH test using SK-MEL-28 cells 48 h after treatment with paclitaxel (PTX). (**A**) Untreated cells, all NADPH-positive, dark spots; (**B**) PTX-treated cells, microcell (red arrow), NADPH-positive cell. The scale bar is 20 μm

To assess the viability of cells after anticancer treatment, we used an NADPH test in vivo. PTX, as an anticancer compound, and the SK-Mel-28 cell line were chosen for this experiment. NADPH test shows the metabolic activity of the cell. Cell’s oxidation processes related to biosynthesis take place in two systems from mitochondria to the endoplasmic reticulum. Hydrogen ion is transferred through the cytochrome system (cytochrome 450) and cell viability is shown [31]. In this experiment, NADPH activity was detected. NADPH is an enzyme that can reduce nitro blue tetrazolium (NBT) dye to the visible reaction product formazan —the reaction involves hydrogen transfer from the substrate, NADPH, to the hydrogen acceptor [25, 32]. The untreated SK-MEL-28 cells exhibited metabolic activity, as expected (Fig. 5A). However, the PTX-treated macrocells had decreased metabolic activity, except for the microcells, which exhibited high NADPH activity (Fig. 5B). Microcells are able to breathe, meaning these cells are not apoptotic and thus cannot die. Consequently, microcellular protein expression is not excluded; this metabolic activity indicates that microcells cells resist the treatment.

### Cell viability detection using NRU

**Fig. 6:**
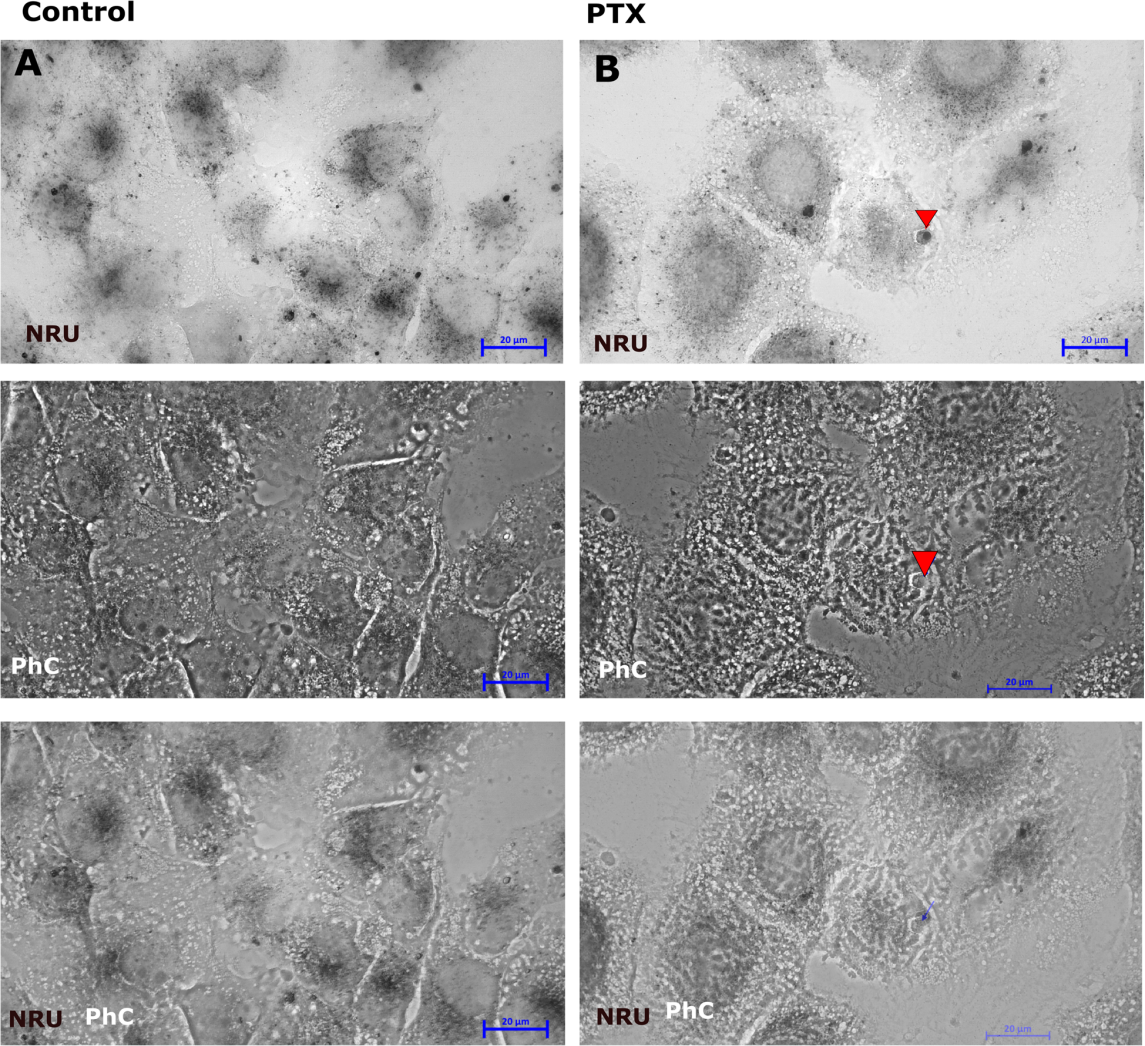
Microcell viability detection using NRU assay in SK-MEL-28 cell line after applied PTX for 48 h. (**A**) Untreated SK-MEL-28 cells with positively NR uptake, NRU – NR uptake, PhC- phase contrasts, NRU/PhC - NRU and PhC overlay; (**B**)SK-MEL-28 cell treated with PTX with significant NR uptake in microcells (NRU, red arrow), NRU – NR uptake, PhC – phase contrast, NRU/PhC - NRU and PhC overlay. Microcell showed intensive NR uptake and this small single cell is localized nearby microcell debris (PhC). The scale bar is 20 μm

Neutral red uptake (NRU) assay is used for cytotoxicity detection. The principle of this assay is based on the detection of viable cells via the uptake of the NRU [33, 34]. The untreated SK-MEL-28 cells (Fig. 6A) presented NR up take via active transport – endocytoses, meaning these cells are viable. Otherwise, PTX treated SK-MEL-28 (Fig. 6B) cell presented lack or no NRU at all, that means cells are non-viable. In turn, the microcell (Fig. 6B, red arrow) presented intensively NR uptake. As it is seen in phase contrast (Fig. 6B, PhC) around the microcell is empty area that indicates to rise endocytosis and cell viability [35].

### Microcells are not expressing TSG101 antigen

**Fig. 7:**
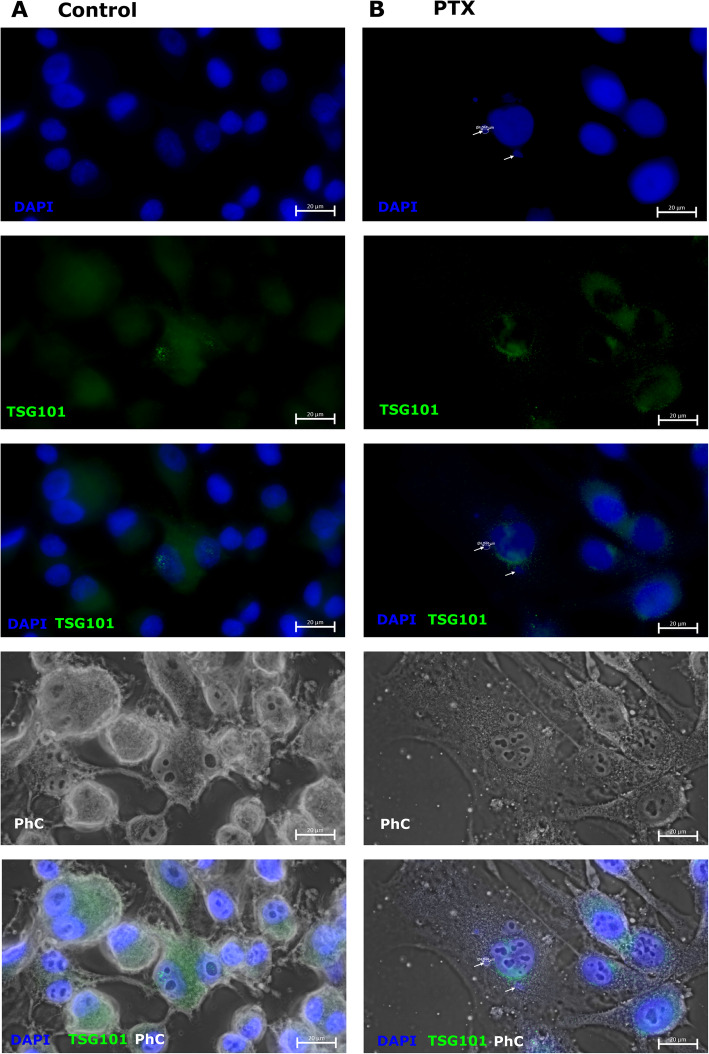
TSG101expression in SK-MEL-28 cell. (**A**) Untreated SK-MEL-28 cells expressed TSG101 (green) marker in their cytoplasm (PhC- phase contrast) localized nearby nucleus (DAPI, blue). Cell nuclei are stained with DAPI (blue) and the last figure in column is overlaid (DAPI/TSG101/PhC); (**B**) Paclitaxel (PTX) treated SK-MEL-28 after 48 h. Cell nuclei are stained with DAPI (blue), cells are captured in phase contrast (PhC) and overlaid (DAPI/TSG101/PhC). After PTX treatment microcell formation was induced (DAPI, blue), nevertheless TSG101 (green) is not expressed in microcells but nearby nuclei. The scale bar is 20 μm

TSG101is a biogenesis factor associated with extracellular vesicles [36]. In this studyTSG101 expression is evaluated in microcells to differentiate microcells from extracellular vesicles. Untreated SK-MEL-28 cells (Fig. 7A) expressed TSG101.The expression of TSG101 is seen as a collection of small green dots (Fig. 7A, TSG101). Antigen is expressed in cell cytoplasm (Fig. 7A, PhC) near the nucleus (Fig. 7A, DAPI/TSG101/PhC). In turn, PTX treatment induced microcell formation in the SK-MEL-28cell line (Fig. 7B, DAPI). Nevertheless TSG101 (Fig. 7B, TSG101) is not expressed in microcells but in the cell cytoplasm (Fig. 7B, PhC) is localized near the nuclei (Fig. 7A, DAPI/TSG101/PhC). This experiment showed that the microcells are not extracellular vesicles which expressing TSG101.

### TEM microcell morphology

**Fig. 8:**
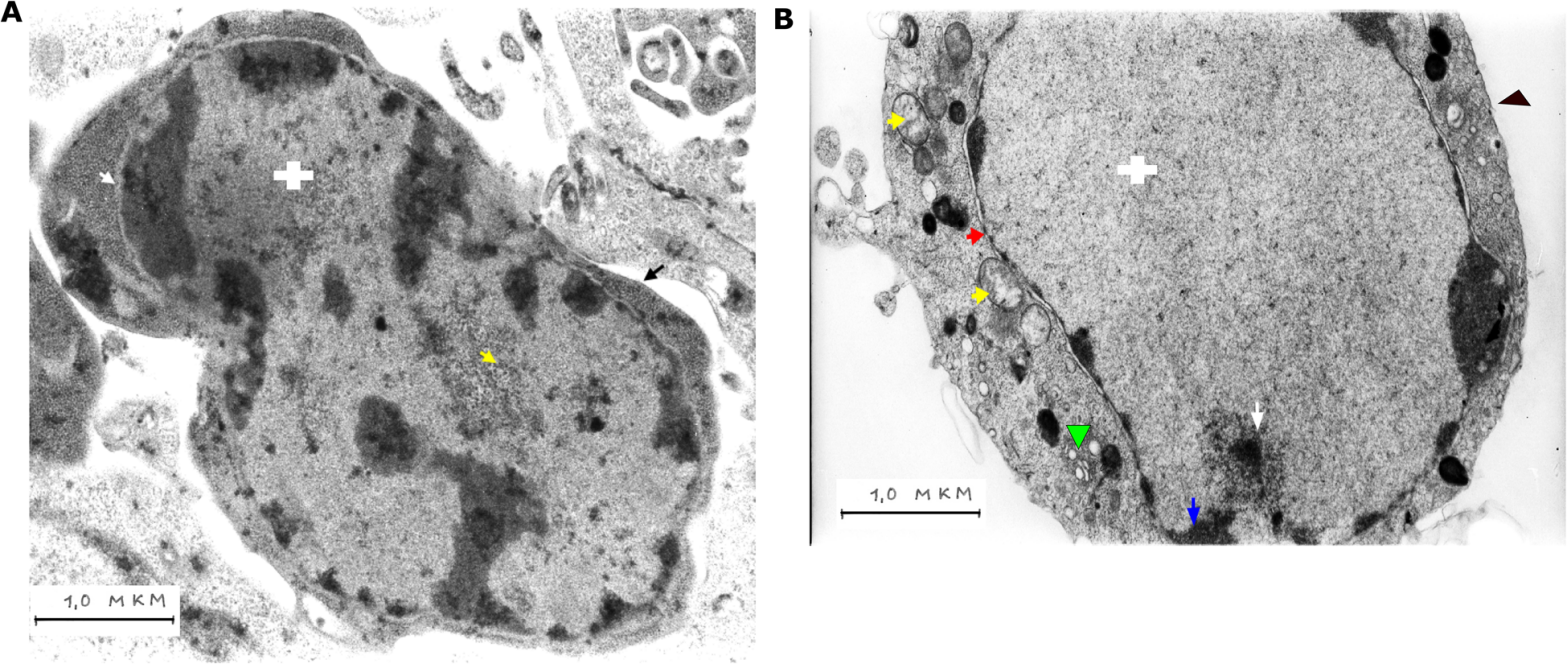
The formed microcell after 48 h Thio-TEPA treatment. (**A**) A microcell with the cell nucleus (white cross), the nuclear envelop (white arrow), the cell membrane (black arrow), nucleolus (yellow arrow). (**B**) A microcell with the cell nucleus (white cross), the nuclear envelop (red arrow), the cell membrane (black arrow head), the heterochromatin (blue arrow), mitochondria (yellow arrows), the Golgi apparatus (green arrow head), nucleoli (white arrow). The scale bar is 1 μm

From our pilot experiments at the ultrastructural level microcell example after Thio-TEPA induction in the Djungarian hamster cell line 4/21 is shown in Fig. 8. It is seen that a microcell is a small cell with an electron-dense cell nucleus, nuclear envelop, cell membranes, cell cytoplasm, and organoids. In the cell cytoplasm are ribosomes, visible as dark spots in the cell cytoplasm. Figure 8A shows a newly formed microcell. It is characterized by the increased relationship between the cell nucleus and the cytoplasm. In the cell cytoplasm are ribosomes, seen as dark spots. The chromatin where heterochromatin, known as inactive chromatin, is part of the cell nucleus is in sufficient quantity suggesting to partly differentiated cell.

In contrast, microcell is shown in Fig. 8B shows increased ratio of the cell nucleus to cytoplasm. Microcell is characterized by the nucleoli and euchromatin, which occupy most of the cell nucleus suggesting to young cell with acquired stem cell properties.

## Discussion

In this work we induced microcells in human cancer cell (SK-MEL-28 and HeLa) lines and human fibroblasts (HS-68) used chemotherapeutic drugs DOX and PTX acting to cells 24 h. We observed that microcells also occur HeLa cells after 6 h 7.5% methanol treatment.

We have showed that microcells develop come into being from apoptotic macrocells, evolving from apoptotic bodies. The microcell formation in this work was observed after cancer cell exposure to chemotherapy or, described by cell morphological changes. Our findings are in agreement with results reported by Buiķis et al. [8] who showed that microcells can form successfully from damaged or fatally damaged macrocells via the sporosis mechanism. Newly developed microcells are independent and mobile, with endocytosis activity. Microcells with a diameter from 2.5 to 5 μm, developed via the sporosis mechanism, could possibly avoid anticancer therapy [37].

Similarly to microcells, Raju cells, nucleolar aggresomes (NoAs), and Bonghan microcells (BH-MCs) have also been observed in cancer cell lines after anticancer treatment. Sundaram et al. have observed the cell formation of mitotic colonies on the monolayer in a cell culture after treatment with etoposide (VP-16) or X-rays [38]. On day 14 after treatment initiation, from the mother cell in a process called neosis, the number of Raju cells raised to approximately 10, with a diameter of 6–10 μM; however, only 8% of them were unable to survive [38]. Raju cells are able to live for 8 weeks after mitotic crisis, after new Raju cell formation from polyploid cells [38]. Another study showed that after treatment with VP-16, NoAs are formed on day five; these NoA cells were shown to contain fibrillarin, rDNA, and pericentric heterochromatin [39]. Unlike microcells and Raju cells, BH-MCs have observed in normal tissues, such as the small intestines and blood cell leukocytes of rats [40]. A BH-MC is a small-sized cell with the ability to divide and pluripotent differencing features, similar to adult stem cells [40]. The differences between microcells and BH-MCs are that microcells are generated from cancer cells, whereas BH-MCs form from micronucleation during normal physiological processes [4, 40].

The differences between microcells, Raju cells, and NoAs are that microcells develop from macrocells 24–48 h after antitumor treatment [4], whereas Raju cells and NoAs develop from the polyploid cell tree up to 7 days after anticancer treatment [8, 38, 39]. Elevated microcell phagocytic ability has observed using carmine red and Indian ink, suggesting their metabolic activity [24]. Microcell formation is a rare process that occurs in less than 1 % of cases [24]. The number of microcells in the population of cell lines considered was small, approximately 1%. Drug-tolerant persister (DTP) cancer cell populations that constitute approximately 0.3–5% of the initial cell population after erlotinib drug are described in other studies [20, 41]. DTP cells are characterized as non-genetic anticancer drug-resistant cells, especially against chemotherapy [20, 41]. Numerous anticancer drugs, such as paclitaxel and doxorubicin, which were used in this study, as well as methanol, cause cell stress, which creates DNA damage and damage to cellular membranes, interrupting cell homeostasis, and inducing apoptosis [42–44]. The cell membrane after treatment with UV radiation and DOX or PTX becomes permeable [43, 45], and it becomes easier to transfect a plasmid DNA into the cells. To avoid damage, cells transform their architecture to a spherical shape during mitosis; this process is controlled by changes in the actin cytoskeleton. However, repairing DNA damage during mitosis is dangerous to cells and could incorrectly affect the results with chromatids separated in anaphases, thus promoting genome instability and cancer genesis [46]. The development and homeostasis in an organism enable apoptosis, a fundamental and complex biological process that destroys unwanted damaged cells [47]. A typical apoptosis scene shows blebbing of the plasma membrane, nuclear fragmentation, chromatin condensation, which includes chromatin parts on the nuclear membrane, and formation of apoptotic bodies [33]. Although an enormous amount of data indicate that cytotoxic drugs induce and activate apoptosis initiation machinery and the cellular stress response, many questions remain unanswered. For instance, the opinion that apoptosis represents the principle of the mechanism by which tumor cells are killed as a result of cancer therapy may not be universally true, as previously proposed by Herr and Debatin [48]. Microcells are small-sized, round or oval cells with small cytoplasm. Microcells increase in count after 24 h of treatment with anticancer drugs; the highest number of microcells was observed 48 h after applying chemotherapy, with the microcells tending to increase in number after chemotherapy, irradiation, or immunotherapy [4, 8]. There is a hypothesis that a microcell forms from a perished macrocell [8]. We showed that in the early stages of microcell formation, cells mainly contain proteins (Fig. 2). ANS solution is a universal and widely used compound for the study of proteins [49]. When microcells are completely formed, nucleic acid is detectable as well (Fig. 3B). The microcell formation under applied therapy is observed in the other research, where is shown that the microcell contain ribosome like particles, nucleus with pronounced functional activity [50]. As it is seen in electron microscopy image (Fig. 8), the microcell has specific organelles characteristic to viable cell. DNA damage, which is caused by oxidative stress, promotes tumorigenesis [51]. The metabolic activity of these cells can be demonstrated by a NADPH test. NADPH performs duties as an antioxidant substrate for the thioredoxin and glutathione antioxidant systems, thereby reducing the level of hypoxia in a cell [18, 52]. Paclitaxel, as an anticancer drug, is not only involved in the hyper-stabilization of microtubules and the inhibition of cytoskeletal restructuration, an increase in metabolic oxidative stress and NADPH oxidase is also associated with paclitaxel’s anticancer effect [53]. NADPH, as a reducing agent, is required for anti-oxidative defense systems—it is a universal electron donor in reductive biosynthesis and detoxification of the cell [15]. We observed a decrease in NADPH activity after anticancer treatment and identified microcells with high NADPH activity. NADPH is produced by metabolically active cells, and this enzyme indicates cell viability [54, 55]. Moreover, this indicates that microcells are metabolically active after PTX treatment. For the NADPH test we used 1 min pre-fixation with 4% formaldehyde solution to reduce activity of NADPH diaphorase activity as NADPH activity in the microcell is very strong. This indicates the rising metabolism of the mircocell after the applied stress. Abid et al. described NADPH oxidase requirement for endothelial cell proliferation and migration activity [56]. NADPH has an essential role in reducing ribonucleotides into deoxyribonucleotides by ribonucleotide reductase; accordingly, it is involved in DNA synthesis by implication [15]. Newly formed microcells are easier to transfect, as they show EGFP expression similarly to other cells. In some research, cancer cells uptake DNA molecules from the cultivation medium to which they are added, more so than non-cancer cells [57, 58]. As described by Kong et al., cancer cell molecule uptake occurs through endocytosis, and this process does not occur in normal cells [57]. In other research, it has been proven that nanoparticles accumulate in cancer microcells, and that microcells have higher endocytosis capability [9]. There are drug-tolerant cancer cells without phenotypic mutation, and these cells are able to proliferate after applying therapy. However, there is a subpopulation that could be a rare mutation causing drug resistance, and these cells can proliferate during anticancer drug effects [19, 59]. In our research, we observed microcell induction, and the number of microcells increased 48 h after applying chemotherapy. In further research, it is important to pay attention to this cell type as a feature of the mechanism of drug resistance or drug-tolerant cells.

## Conclusions

Herein, we characterized microcells morphologically. The number of microcells increased following the application of therapy, and these cells were natural components of tumors. Microcells are a subpopulation of cancer characterized by anticancer drug resistance. Therefore, analysis of the microcell population in a tumor undergoing anticancer treatment could be a strong prognostic factor for a patient’s survival, and could be a potential regenerator of cancer cells after the death of the tumor itself.

## Data Availability

The relevant data supporting the conclusions of this article are included within the article and its additional files.
